# GenAI-supported co-creation among humanities undergraduates: Offloading, agency, and future task confidence in a stimulus-organism-response framework

**DOI:** 10.1371/journal.pone.0355282

**Published:** 2026-09-02

**Authors:** XiaCheng Song, Lu Sun, Huafeng Qu, Xiaojun Jiang, Jing Jin, Lulu Feng, Wenwen Song

**Affiliations:** 1 Xi’an Fanyi University, Xi’an, China; 2 Yunnan College of Business Management, Anning, Kunming, Yunnan, China; 3 Universiti Kebangsaan Malaysia, Bangi, Selangor, Malaysia; 4 Guangdong University of Science and Technology, Dongguan, China; 5 Panzhihua University, Panzhihua, China; 6 Xidian University, Xi’an, China; Hunan Normal University, CHINA

## Abstract

This study examines how humanities undergraduates’ perceptions of GenAI interaction features relate to co-creation and future task confidence within a stimulus-organism-response framework. Drawing on survey data from 517 Chinese university students, we conducted observed-variable path analysis and bootstrap mediation analysis. The revised model allowed residual correlations among organism-level states measured in the same survey session and showed good fit (chi-square = 3.56, df = 2, CFI = .999, TLI = .987, RMSEA = .039, SRMR = .008). Perceived GenAI interaction features were positively associated with beneficial offloading, dependent offloading, agency/autonomy, and self-efficacy. Beneficial offloading, agency/autonomy, and self-efficacy were positively associated with both co-creation and future task confidence, whereas dependent offloading was not significantly associated with either outcome after the other organism variables were considered. Bootstrap analyses indicated significant indirect associations through beneficial offloading, agency/autonomy, and self-efficacy, but not through dependent offloading. Harman’s single-factor test indicated that the first unrotated factor explained 35.2% of the variance, below the conventional 50% threshold. Sensitivity analyses further examined a co-creation-to-future-confidence path and disaggregated stimulus features. The findings suggest that humanities students’ reported GenAI-supported co-creation and future task confidence are linked less to offloading in general than to productive offloading, agency, and confidence-supportive use.

## 1. Introduction

GenAI tools have moved rapidly from experimental gadgets to everyday companions in university learning, especially in language and humanities subjects [1–3]. Students now draft, revise, translate, and critique texts with the assistance of conversational systems that can generate fluent language in seconds [4–6]. Public and institutional debates, however, tend to oscillate between enthusiasm about productivity gains and anxiety about plagiarism, academic integrity, and skill erosion [6,7]. In both narratives, students are largely portrayed as either beneficiaries or offenders, rather than as active agents who strategically integrate GenAI into their learning practices [8–10]. If research remains at this level of abstraction, it cannot explain how students actually collaborate with GenAI in concrete tasks, nor can it guide educators in designing responsible forms of GenAI supported learning [6]. In disciplines such as languages, literature, and broader humanities fields, students spend much of their time reading, interpreting, and producing text, which makes GenAI tools especially attractive as collaborators and raises distinctive questions about how such students integrate GenAI into their core disciplinary work [11,12].

Empirical work on GenAI in higher education is only beginning to catch up with this reality, and much of it adopts classic technology acceptance perspectives [13]. Studies commonly focus on perceived usefulness, ease of use, attitudes, and behavioral intention, or on outcome comparisons between users and non-users in terms of grades or writing quality [14,15]. This line of work is informative, yet it treats GenAI mainly as a neutral tool that is either adopted or rejected. It rarely examines what students choose to offload to GenAI, what they insist on doing themselves, and how these choices relate to their sense of control and capability [16–18]. Moreover, students are often treated as a homogeneous group, even though their actual ways of integrating GenAI into academic work may vary considerably. Without a closer look at these internal processes, it remains unclear whether GenAI amplifies or undermines learning in actual academic settings [16–18].

Against this backdrop, the study addresses the following research questions:

RQ1. How are perceived GenAI interaction features related to students’ co-creation experiences with GenAI and to their future task confidence in GenAI-assisted language learning?

**RQ2.** To what extent are these relationships transmitted through beneficial offloading, dependent offloading, agency/autonomy, and self-efficacy?

RQ3. How do the specific indirect associations via beneficial offloading, dependent offloading, agency/autonomy, and self-efficacy compare in relation to co-creation and future task confidence?

## 2. Theoretical framework and literature review

The present study approaches this problem through a stimulus–organism–response lens that foregrounds psychological mechanisms rather than surface adoption [17]. In this view, perceived interaction features of GenAI systems, including anthropomorphism and social presence, explainability, and competence trust, function as stimuli that shape students’ internal states [19,20].

### 2.1. Stimulus: GenAI interaction features

GenAI is experienced as an interactive partner rather than a static tool, so perceived interaction features can function as “stimuli” in an S–O–R process [21,22]. We focus on anthropomorphism/social presence, explainability, and competence trust because they represent complementary facets of the same perceived human-GenAI interaction environment: anthropomorphism and social presence capture the relational quality of the interaction, explainability captures whether system responses are understandable enough to support student control, and competence trust captures whether the system is perceived as capable enough to be used in academic work. In the main S-O-R model, these facets were therefore treated as a composite stimulus rather than as separate competing mechanisms; the disaggregation analysis reported below was used to check whether this parsimonious treatment obscured substantively different patterns.

### 2.2. Cognitive offloading: beneficial vs dependent

We distinguish beneficial offloading, where GenAI takes over routine or mechanical aspects of work and frees cognitive resources, from dependent offloading, where students hand over key steps before engaging with the task themselves [23,24].

### 2.3. Organism states: learner agency/autonomy and self-efficacy

These internal states include not only general trust or satisfaction, but also more specific forms of cognitive offloading and agency. Alongside these offloading strategies, we examine students’ sense of agency or autonomy when working with GenAI and their self-efficacy in handling GenAI supported language tasks. The central claim is that these organism level constructs jointly determine whether students experience collaboration with GenAI as genuinely productive and whether they intend to continue using GenAI in a reflective way [10,25,26].

### 2.4. Response outcomes: co-creation and future task confidence

As response outcomes, co-creation captures students’ perceived synergy and value when collaborating with GenAI in authentic writing and interpretation tasks, whereas future task confidence reflects students’ confidence in completing similar writing tasks in the future. This label follows the wording of the observed item used in the present analysis and is therefore more precise than the earlier response-outcome label. Both outcomes were expected to be associated with organism-level regulation (beneficial versus dependent offloading) and learners’ perceived control and capability (agency/autonomy and self-efficacy).

### 2.5 Summary and conceptual model

Taken together, the above arguments support a stimulus-organism-response framework in which perceived GenAI interaction features are linked to co-creation and future task confidence through multiple organism-level mechanisms. In the present study, beneficial offloading, dependent offloading, agency/autonomy, and self-efficacy are conceptualized as distinct organism states linking perceived interaction features to downstream responses. Based on this framework, the study examines both direct associations among these variables and indirect pathways through which perceived GenAI interaction features are statistically related to students’ co-creation and future task confidence.

## 3. Methods

### 3.1. Research context and design

This study formed part of a larger project on GenAI-assisted language learning in mainland China and relied on a cross-sectional survey design. The focal setting was university-level academic writing and language use, where students routinely consult large language models for translation, proofreading, and content development. Guided by a stimulus-organism-response framework, we conceptualized perceived interaction features of the focal GenAI tool as the stimulus, multiple psychological states related to cognitive offloading and agency as the organism, and co-creation experiences and future task confidence as the responses.

### 3.2. Participants

Participants were 517 undergraduate students enrolled in language and other humanities programs at a comprehensive university in mainland China. All were active users of GenAI tools in their study or writing activities and completed the questionnaire voluntarily during regular class sessions or through course-related online channels. Responses were screened using an attention check and completion-time criteria; no cases met the exclusion thresholds, and all 517 responses were retained for analysis. The sample was predominantly female, with roughly four fifths of respondents identifying as women, and most participants were in their first or second year of study. Students from science, engineering, medicine, and other quantitatively oriented disciplines were not included, in order to concentrate on majors where text production and interpretation constitute the core of academic work.

### 3.3. Measures

All focal constructs were measured using items adapted from a previously validated questionnaire on GenAI-assisted language learning and from a related scale-development manuscript based on the same dataset. Perceived GenAI interaction features (Stimulus, S) captured students’ perceptions of their most frequently used GenAI tool as humanlike, socially present, explainable, and trustworthy, assessed with six Likert-type items. Beneficial offloading (BO) and dependent offloading (DO) were each measured with three items, reflecting, respectively, the extent to which delegating routine language work to GenAI freed cognitive resources for higher-order thinking versus a tendency to rely on GenAI for demanding tasks without first attempting them independently. Agency/autonomy (AG) was assessed with three items indexing students’ perceived decision-making control when using GenAI as a tool rather than a controller. Unless otherwise noted, items used a five-point agreement scale (1 = strongly disagree to 5 = strongly agree).

Three additional constructs were modeled as separate observed variables. Co-creation (CC) indexed the perceived added value of ‘me + AI’ collaboration relative to either party working alone. Future task confidence (FTC) captured students’ confidence in completing similar writing tasks in the future and was measured with Q27 (‘In the future, when I encounter similar writing tasks, I will be more confident in completing them’). Self-efficacy (SE) was specified as an organism-level mediator and reflected confidence-related willingness to attempt foreign-language or academic-language expression with AI support. Consistent with the measurement specification in the related validation study, SE, CC, and FTC were each assessed with a single item; therefore, internal-consistency estimates are not applicable for these variables, and this measurement choice is treated as a limitation.

For transparency, the core item mapping was as follows: S used Q2, Q3, Q4, Q6, Q7, and Q9; BO used Q10, Q12, and Q13; DO used Q15, Q16, and Q18; AG used Q19, Q21, and Q22; CC used Q24; SE used Q25; and FTC used Q27. The anonymized dataset and codebook supplied as Supporting Information provide the complete item wording and coding.

### 3.4. Procedure and ethical considerations

Data were collected through an anonymous online questionnaire administered via Wenjuanxing (Questionnaire Star) during regular course periods. Participant recruitment and data collection took place in November 2025. After following a link distributed by instructors or through course messaging channels, students first viewed an information sheet explaining the study purpose, the voluntary nature of participation, anonymity, confidentiality, and data-protection procedures. Electronic informed consent was obtained before respondents could access the questionnaire. The study protocol, recruitment procedures, and consent materials were reviewed and approved by Xi’an Fanyi University (Research Office) (approval no. XFU-ETH-2025–1125; approval date: 25 November 2025). All procedures complied with institutional guidelines for research involving human participants and with the principles of the Declaration of Helsinki.

### 3.5. Data analysis

All analyses were conducted in R using the lavaan package. Descriptive statistics, Cronbach’s alpha coefficients, and zero-order correlations were first computed for the study variables. For the multi-item constructs--stimulus (S), beneficial offloading (BO), dependent offloading (DO), and agency/autonomy (AG)--composite scores were calculated by averaging the corresponding items. Self-efficacy (SE), co-creation (CC), and future task confidence (FTC) were treated as observed single-item variables.

To examine the hypothesized S-O-R relationships, we conducted observed-variable path analysis with robust maximum likelihood estimation (MLR). This approach was chosen instead of a latent-variable SEM for pragmatic and methodological reasons: the study tested structural associations among construct scores rather than revalidating the measurement model, several response variables were measured with single items, and a parallel validation manuscript already focuses on the fuller measurement evidence [27]. Observed-variable path analysis therefore provided a transparent way to estimate the proposed S-O-R associations while avoiding a mixed latent-observed model that would give uneven treatment to multi-item and single-item constructs. Because BO, DO, AG, and SE were organism-level states measured in the same survey session and were theoretically expected to covary beyond the common stimulus variable, the revised main model allowed residual correlations among these organism variables. The model also allowed the residuals of CC and FTC to covary. Standardized direct effects were estimated for paths from stimulus to organism variables and from organism variables to response outcomes. Indirect associations were estimated using bootstrap resampling with 5,000 samples; an indirect association was considered statistically significant when its 95% confidence interval did not include zero [28].

Common method variance was examined using Harman’s single-factor test based on the 18 Likert-type items. To address model-specification concerns, we also estimated two sensitivity analyses: an alternative cross-sectional model adding a CC - > FTC path, and a stimulus-disaggregation model separating anthropomorphism/social presence (Q2 and Q4), explainability (Q3), and competence/trust (Q6, Q7, and Q9). These analyses were used to evaluate whether the main interpretation depended on treating stimulus features as a single composite.

## 4. Results

### 4.1. Sample characteristics and correlations among key constructs

Table 1 presents descriptive statistics and zero-order correlations among the focal constructs. The multi-item measures showed acceptable internal consistency (Cronbach’s alpha = .72−.80), and the means were generally above the scale midpoint (Ms = 3.05–3.64; SDs = 0.54–0.79). The correlation pattern was broadly consistent with the proposed S-O-R framework: the stimulus construct (S) was positively associated with each organism variable (BO r = .67; DO r = .33; AG r = .36; SE r = .44) and with both responses (CC r = .36; FTC r = .45). Beneficial offloading, agency/autonomy, and self-efficacy were moderately correlated with both co-creation and future task confidence, whereas dependent offloading showed comparatively weak correlations with the two responses.

**Table 1:** Descriptive statistics and zero-order correlations among study constructs.

Construct	α	M	SD	1	2	3	4	5	6	7
1. Stimulus (S)	0.80	3.30	0.54	1.00	0.67	0.33	0.36	0.44	0.36	0.45
2. Beneficial offloading (BO)	0.77	3.54	0.58	0.67	1.00	0.35	0.53	0.46	0.49	0.56
3. Dependent offloading (DO)	0.72	3.05	0.62	0.33	0.35	1.00	0.10	0.11	0.13	0.14
4. Agency/autonomy (AG)	0.75	3.64	0.62	0.36	0.53	0.10	1.00	0.46	0.51	0.54
5. Self-efficacy (SE)	—	3.41	0.68	0.44	0.46	0.11	0.46	1.00	0.49	0.51
6. Co-creation (CC)	—	3.58	0.79	0.36	0.49	0.13	0.51	0.49	1.00	0.51
7. Future task confidence (FTC)	—	3.51	0.68	0.45	0.56	0.14	0.54	0.51	0.51	1.00

Note. alpha = Cronbach’s alpha. M = mean; SD = standard deviation. Zero-order correlations are Pearson correlations. For single-item measures (SE, CC, and FTC), internal consistency reliability is not applicable (reported as ‘--’).

### 4.2. Theoretical S–O–R framework

**Fig 1:**
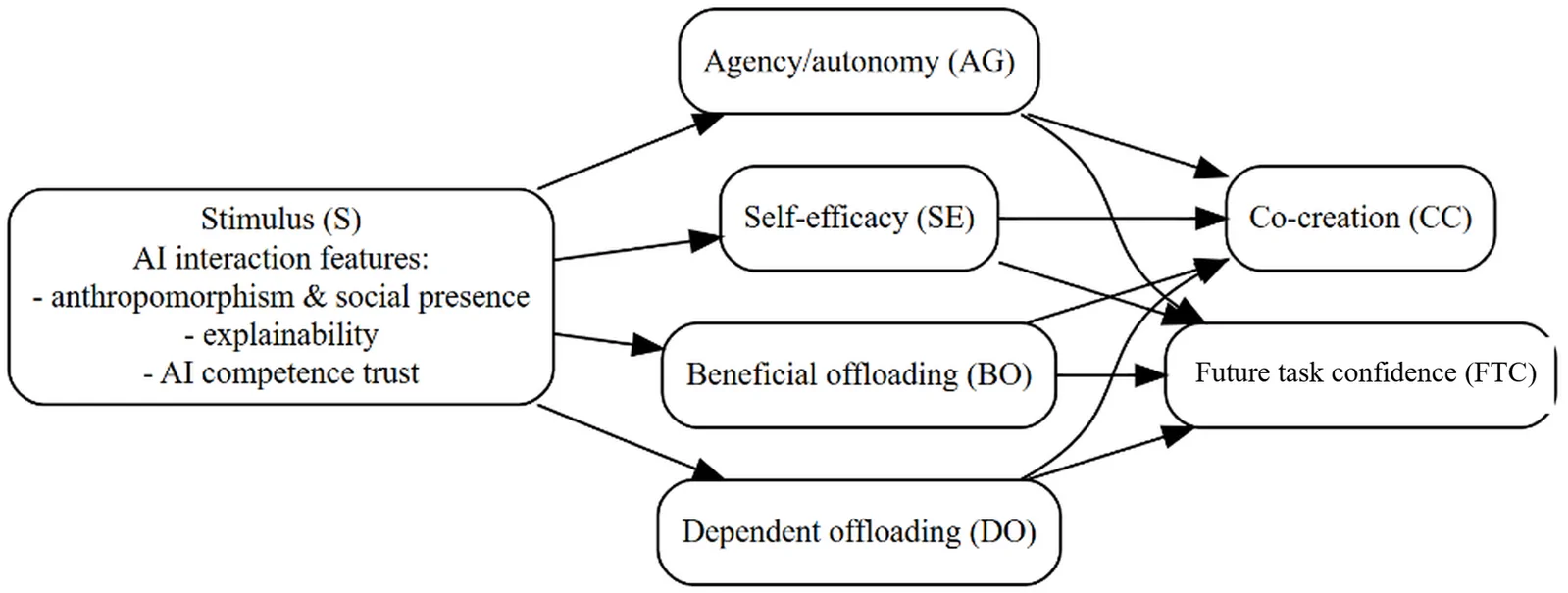
Theoretical S–O–R framework for GenAI-assisted language learning. Note. S = stimulus (AI interaction features, including anthropomorphism, social presence, explainability, and AI competence trust); BO = beneficial offloading; DO = dependent offloading; AG = agency/autonomy; SE = self-efficacy; CC = co-creation; FTC = future task confidence.

Fig 1 presents the theoretical stimulus-organism-response (S-O-R) framework guiding the present study. Stimulus (S) represents perceived GenAI interaction features, including anthropomorphism, social presence, explainability, and competence-based trust. These perceived features are hypothesized to be associated with four organism-level states: beneficial offloading (BO), dependent offloading (DO), agency/autonomy (AG), and self-efficacy (SE). In turn, these organism variables are expected to be associated with two response outcomes, namely co-creation (CC) and future task confidence (FTC).

This figure illustrates the theoretical stimulus-organism-response (S-O-R) framework guiding the present study and is not based on statistical estimation.

### 4.3. Direct effects in the observed-variable path analysis

To examine the hypothesized S-O-R relationships, an observed-variable path analysis was conducted using composite scores for the multi-item constructs and single-item observed indicators for SE, CC, and FTC. The revised main model, which allowed residual correlations among the organism-level variables, showed good fit: chi-square = 3.56, df = 2, CFI = .999, TLI = .987, RMSEA = .039, and SRMR = .008. Table 2 presents the standardized direct effects.

**Table 2:** Standardized direct effects in the observed-variable path analysis.

From	To	β	SE	z	p
S	BO	0.666	0.026	25.200	<.001
S	DO	0.330	0.054	6.154	<.001
S	AG	0.359	0.048	7.478	<.001
S	SE	0.444	0.047	9.387	<.001
BO	CC	0.225	0.053	4.240	<.001
DO	CC	−0.001	0.042	−0.032	.975
AG	CC	0.277	0.051	5.440	<.001
SE	CC	0.260	0.059	4.420	<.001
BO	FTC	0.324	0.049	6.610	<.001
DO	FTC	−0.024	0.046	−0.521	.602
AG	FTC	0.258	0.052	5.000	<.001
SE	FTC	0.243	0.051	4.750	<.001

Note. All coefficients are standardized estimates from the observed-variable path analysis with correlated residuals among organism-level variables. S = stimulus; BO = beneficial offloading; DO = dependent offloading; AG = agency/autonomy; SE = self-efficacy; CC = co-creation; FTC = future task confidence.

As shown in Table 2, perceived GenAI interaction features were positively associated with all four organism variables. Specifically, stimulus was associated with beneficial offloading (beta = .666, p < .001), dependent offloading (beta = .330, p < .001), agency/autonomy (beta = .359, p < .001), and self-efficacy (beta = .444, p < .001). These results indicate that students who perceived stronger interaction qualities in GenAI also reported stronger cognitive and motivational responses to its use.

At the organism-to-response stage, beneficial offloading, agency/autonomy, and self-efficacy each showed significant positive associations with both co-creation and future task confidence. Beneficial offloading was associated with co-creation (beta = .225, p < .001) and future task confidence (beta = .324, p < .001). Agency/autonomy was also associated with co-creation (beta = .277, p < .001) and future task confidence (beta = .258, p < .001), as was self-efficacy (beta = .260, p < .001 for co-creation; beta = .243, p < .001 for future task confidence). In contrast, dependent offloading was not significantly associated with co-creation (beta = −.001, p = .975) or future task confidence (beta = −.024, p = .602). These findings indicate that the organism-level variables were not equivalent in their associations with the two response outcomes.

### 4.4. Indirect effects via bootstrap mediation

To examine the proposed mediating pattern within the limits of the cross-sectional design, bootstrap analyses with 5,000 resamples were conducted to estimate specific and total indirect associations from stimulus to co-creation and future task confidence through BO, DO, AG, and SE (Table 3). For co-creation, significant indirect associations were observed through beneficial offloading (beta = .150, p < .001, 95% CI [.078,.222]), agency/autonomy (beta = .099, p < .001, 95% CI [.057,.141]), and self-efficacy (beta = .116, p < .001, 95% CI [.059,.173]), whereas the indirect association through dependent offloading was not significant (beta = .000, p = .975, 95% CI [−.028,.027]). The total indirect association from stimulus to co-creation was significant (beta = .364, p < .001, 95% CI [.293,.435]). A similar pattern emerged for future task confidence: significant indirect associations were found through beneficial offloading (beta = .216, p < .001, 95% CI [.147,.285]), agency/autonomy (beta = .093, p < .001, 95% CI [.048,.137]), and self-efficacy (beta = .108, p < .001, 95% CI [.054,.162]), whereas the indirect association through dependent offloading remained non-significant (beta = −.008, p = .597, 95% CI [−.038,.022]). The total indirect association from stimulus to future task confidence was also significant (beta = .409, p < .001, 95% CI [.328,.489]).

**Table 3:** Standardized indirect effects of S on co-creation and future task confidence based on 5,000 bootstrap resamples.

Path	Mediator	β	SE	z	p	95% CI
S - > CC	BO	0.150	0.037	4.080	<.001	[0.078, 0.222]
S - > CC	DO	0.000	0.014	−0.032	.975	[-0.028, 0.027]
S - > CC	AG	0.099	0.021	4.650	<.001	[0.057, 0.141]
S - > CC	SE	0.116	0.029	3.970	<.001	[0.059, 0.173]
S - > CC	Total	0.364	0.036	10.000	<.001	[0.293, 0.435]
S - > FTC	BO	0.216	0.035	6.120	<.001	[0.147, 0.285]
S - > FTC	DO	−0.008	0.015	−0.529	.597	[-0.038, 0.022]
S - > FTC	AG	0.093	0.023	4.100	<.001	[0.048, 0.137]
S - > FTC	SE	0.108	0.028	3.920	<.001	[0.054, 0.162]
S - > FTC	Total	0.409	0.041	9.900	<.001	[0.328, 0.489]

Note. BO = beneficial offloading; DO = dependent offloading; AG = agency/autonomy; SE = self-efficacy; CC = co-creation; FTC = future task confidence. Standardized indirect effects were estimated using bootstrap resampling (5,000 samples). Confidence intervals not including zero indicate significant indirect effects.

### 4.5. Common method variance and sensitivity analyses

Harman’s single-factor test was conducted using the 18 Likert-type items. The first unrotated factor explained 35.2% of the total variance, below the conventional 50% threshold, suggesting that a single common factor did not dominate the item covariance structure. This result does not eliminate the possibility of common method variance, but it provides a diagnostic check requested during review.

We also estimated an alternative cross-sectional model including a CC - > FTC path to examine whether co-creation was statistically associated with future task confidence. This model showed good fit (chi-square = 3.56, df = 2, CFI = .999, TLI = .987, RMSEA = .039, SRMR = .008). The CC - > FTC path was significant (beta = .170, p = .004), while BO, AG, and SE remained positively associated with FTC and DO remained non-significant. Thus, the added path did not change the main interpretation that beneficial offloading, agency/autonomy, and self-efficacy were the reliable organism-level correlates of the response outcomes.

Finally, we disaggregated the stimulus construct into anthropomorphism/social presence, explainability, and competence/trust. The disaggregated model showed good fit (chi-square = 13.5, df = 6, CFI = .994, TLI = .970, RMSEA = .049, SRMR = .016). The pattern suggested that competence/trust was most consistently associated with BO, DO, and SE, whereas explainability was especially associated with AG and SE. The organism-to-response paths remained substantively unchanged. These results support the use of the composite stimulus score for the main S-O-R model while acknowledging that specific interaction features may operate through partially different psychological routes.

## 5. Discussion

### 5.1. Summary of main findings

The present study examined how humanities undergraduates’ perceptions of GenAI interaction features were associated with co-creation experiences and future task confidence within a stimulus-organism-response framework. Perceived GenAI interaction features were positively associated with all four organism variables, namely beneficial offloading, dependent offloading, agency/autonomy, and self-efficacy. However, these organism states were not associated with downstream responses in the same way. Beneficial offloading, agency/autonomy, and self-efficacy consistently showed positive direct and indirect associations with both co-creation and future task confidence. In contrast, dependent offloading displayed small and non-significant direct and indirect associations in the revised model.

### 5.2. Pathways linking GenAI interaction features to co-creation and future task confidence

The findings support treating perceived GenAI interaction features as a meaningful stimulus within the S-O-R framework, while also showing that this stimulus is not a single undifferentiated mechanism. When students experienced GenAI as socially present, humanlike, transparent, and competent, they tended to report higher beneficial offloading, agency/autonomy, and self-efficacy [29–31]. The stimulus-disaggregation analysis further suggested that explainability and competence/trust may be particularly relevant to different organism-level responses. These patterns should be interpreted as cross-sectional associations rather than causal effects.

### 5.3. Distinct roles of beneficial versus dependent offloading and learner agency

A central contribution of this study is that it distinguishes beneficial from dependent offloading and evaluates their roles alongside learner agency and self-efficacy. Beneficial offloading, agency/autonomy, and self-efficacy showed consistently positive links to both co-creation and future task confidence, suggesting that GenAI may be most productively integrated when it reduces routine workload while preserving students’ perceived control and competence [32–35]. In contrast, dependent offloading showed small, non-significant associations with both outcomes in this sample. This should be read cautiously: the null pattern does not demonstrate that dependent offloading is harmless, nor does it address objective learning quality or longer-term skill development [36–39]. Rather, it indicates that dependent offloading was not a reliable correlate of students’ reported co-creation or future task confidence once the other organism variables were considered.

### 5.4. Limitations and directions for future research

Interpretation of these findings should consider several limitations. First, the data were drawn from a cross-sectional self-report survey, which limits causal inference, leaves open reciprocal relations, and does not rule out common method variance even though Harman’s single-factor test did not indicate a dominant single factor. The path coefficients and indirect associations should therefore be read as statistical associations consistent with the S-O-R framework, not as evidence that perceived stimulus features caused later organism states or response outcomes. Second, the sample comprised undergraduates from a single Chinese university, was approximately 78% female, and included active GenAI users; this limits generalizability to other academic fields, institutions, national contexts, and students with little or no GenAI experience. Third, the present study employed observed-variable path analysis based on composite scores for multi-item constructs and single-item indicators for SE, CC, and FTC. This approach is transparent and parsimonious, but it cannot isolate measurement error for the single-item constructs or provide internal-consistency reliability estimates for them. Fourth, the outcomes were subjective perceptions rather than objective indicators of writing quality, learning performance, or actual long-term tool use. Future research should use longitudinal, experimental, or mixed-method designs; include multi-item measures of response outcomes; and integrate objective indicators of learning quality and GenAI-use behavior.

## 6. Conclusion

This study applied a stimulus-organism-response framework to examine how perceived GenAI interaction features are associated with humanities undergraduates’ co-creation with GenAI and their future task confidence in text-centered learning. The findings indicate that perceived interaction features are statistically linked to both responses primarily through organism-level states, especially beneficial offloading, agency/autonomy, and self-efficacy. By contrast, dependent offloading showed no meaningful direct or indirect association with co-creation or future task confidence once the other organism variables were considered. These results suggest that not all forms of GenAI-enabled offloading are equally productive in students’ perceptions. Within the limits of a cross-sectional single-institution survey, the study highlights the importance of supporting forms of GenAI use that reduce routine burden while preserving learner agency and confidence.

## Supporting information

S1 Dataset Anonymized minimal dataset used to reproduce the reported analyses. (CSV)

S1 Codebook Variable codebook for the anonymized minimal dataset. (CSV)

S1 Script R analysis script used for the revised model estimation and reported analyses. (R)
